# Internet of Things (IoT)-Based Environmental Monitoring and Control System for Home-Based Mushroom Cultivation

**DOI:** 10.3390/bios13010098

**Published:** 2023-01-06

**Authors:** Jiu Li Chong, Kit Wayne Chew, Angela Paul Peter, Huong Yong Ting, Pau Loke Show

**Affiliations:** 1 Zhejiang Provincial Key Laboratory for Subtropical Water Environment and Marine Biological Resources Protection, Wenzhou University, Wenzhou 325035, China; 2 Department of Chemical and Environmental Engineering, Faculty of Science and Engineering, University of Nottingham, Jalan Broga, Semenyih 43500, Selangor, Malaysia; 3 School of Chemistry, Chemical Engineering and Biotechnology, Nanyang Technological University, 62 Nanyang Drive, Singapore 637459, Singapore; 4 Postgraduate Studies Unit, Research and Postgraduate Centre, Xiamen University Malaysia, Jalan Sunsuria, Bandar Sunsuria, Sepang 43900, Selangor, Malaysia; 5 Drone Research and Application Centre, University of Technology Sarawak, No. 1, Jalan Universiti, Sibu 96000, Sarawak, Malaysia; 6 Department of Sustainable Engineering, Saveetha School of Engineering, SIMATS, Chennai 602105, India

**Keywords:** Internet of Things (IoT), cultivation, mushroom, environmental control, remote monitoring

## Abstract

The control and monitoring of the environmental conditions in mushroom cultivation has been a challenge in the mushroom industry. Currently, research has been conducted to implement successful remote environmental monitoring, or, in some cases, remote environmental control, yet there is not yet a combination of both these systems providing live stream images or video. As a result, this research aimed to design and develop an Internet of things (IoT)-based environmental control and monitoring system for mushroom cultivation, whereby the growth conditions of the mushrooms, such as temperature, humidity, light intensity, and soil moisture level, are remotely monitored and controlled through a mobile and web application. Users would be able to visualize the growth of the mushroom remotely by video and images through the Internet. The respective sensors are implemented into the mushroom cultivation process and connected to the NodeMCU microcontroller, which collects and transfers the data to the cloud server, enabling remote access at any time through the end device with internet connection. The control algorithm regulates the equipment within the cultivational chamber autonomously, based on feedback from the sensors, in order to retain the optimum environment for the cultivation of mushrooms. The sensors were tested and compared with manual readings to ensure their accuracy. The implementation of IoT toward mushroom cultivation would greatly contribute to the advancement of the current mushroom industry which still applies the traditional cultivation approach.

## 1. Introduction

Industry revolution 4.0 nowadays incorporates the digital transformation of the entire industrial and consumer markets [1]. Digitalization has become an appealing characteristic and a trend for different industries, especially the implementation of the Internet of Things (IoT). In this paper, research was carried out to develop an automated environmental control and monitoring system for home-based mushroom cultivation. Food is one of the necessities for life and should be supplied in good quality and quantity. However, humanity has struggled with food shortage for millennia, owing to inadequate agricultural production, and it is becoming more severe, due to rising population, urbanization, and desertification [2]. In addition, the agricultural sector confronts a significant challenge in expanding food production by 60% to supply the estimated world population of 9.3 billion of 2050, while coping with the greater intensity and higher frequency of extreme weather events [3]. As a result, urban farming is gaining prominence as an agro-economic activity, due to its potential in utilizing limited space, alleviating food shortages, and increasing the income of local communities [4]. The cultivation of mushrooms has gained a lot of attention as mushrooms do not require cultivable land, capable of being cultivated on shelves in indoor space. Cultivation of mushrooms is also an eco-friendly and profitable process that can provide fast yielding and nutritious sources of food [5].

Mushrooms are the spore-bearing fruiting bodies or the sporocarps of certain fungi [6]. Edible mushrooms are full of nutritional components, such as proteins, amino acids, and vitamins, as well as other functional substances, namely, polysaccharides, nucleic acids, and polyphenols. Nucleic acid components have preventive functions against human lifestyle-related diseases, such as diabetes and hyperlipidaemia, while enhancing the human immune system [7]. Hence, with rising awareness of the scientific diet and increasing living standards, the mushroom market has steadily grown in recent years. Cultivation of mushrooms has become a trend and a means for future rapid expansion of the mushroom industry [8].

Despite improvements in mushroom cultivation methods over time to cope with increasing demand, such as substrate sterilization, spawn preparation and pathogen control, the lack of automated systems, to provide warnings when significant changes in environmental conditions are present in the mushroom cultivation chamber, may inhibit the growth of the mushrooms. The growth process of the mushroom involves mycelium development and fruiting, where the growth of the mushroom is vulnerable for a period for weeks [9]. The major ecological parameters that affect the yield, stalk height, stalk diameter and capsize of the mushroom are temperature, humidity, carbon dioxide level, and soil moisture level [10]. Different growth stages of the mushroom require different environmental conditions, and they vary between different species of mushrooms [11]. For instance, during the fruiting stage for the white button mushroom (Agaricus Bisporus), the temperature should be maintained at 15 °C to 22 °C, the relative humidity should be controlled at 85% to 90% RH and the concentration of carbon dioxide should be kept below 1000 μmol/mol [9]. High temperature, excess humidity and low aeration rate should be avoided during the cultivation process, as they may result in various viral infections that inhibit the growth of mycelia and damage the fruiting mechanism, resulting in a lower yield of mushrooms [12]. As a result, implementing a monitoring and control-based network of plug-and-play sensors and Internet of things (IoT) systems into mushroom farming processes could lower costs through automation and allow users to monitor mushroom growth and real-time conditions from anywhere. Furthermore, with the control system in place, users are able to obtain a higher yield of the mushroom by providing the optimum growth conditions [13].

The Internet of things (IoT) could be defined as a network for the implementation of independent federated services and applications characterized by a high degree of autonomous data gathering, event transfer, network connectivity, decision making, responding to feedback, and interoperability [14]. The internet develops the integrated network while the things integrate the generic object into an easy access framework. In other words, the IoT provides a new platform that connects computing devices, mechanical or digital machinery objects, animals, and people with unique identities (UIDs) for data exchange without the necessity of human-to-computer or human-to-human interaction [15]. The IoT primarily relies on the wireless sensor network (WSN), which is a network of nodes that can collect, transmit and process data around its periphery, hence providing the possibility to solve problems, and to initiate and govern machine-to-machine interactions [16]. Moreover, to achieve the objective of minimum human intervention while using the IoT, the targeted objects are equipped with microcontrollers as well as transceivers to allow configuration and communication with the protocol stacks that detect the interactions between the objects [17]. However, with the increasing number and varieties of connected devices in the IoT network, the challenges of object identification, data management, mining and security have risen. These challenges can be resolved by incorporating security solutions, such as firewalls, and standardizing and lowering the complexity of the system [18]. In addition, there are various types of development boards that enable the prototypes of new IoT projects, namely, microcontroller boards, System on Chip (SoC) boards and the Single-board Computer (SBC). The Node Microcontroller Unit (NodeMCU) and Raspberry Pi 4B SBC were selected for this research. The NodeMCU is a budget, open-source firmware, based on the ESP8266 Wi-Fi SoC, where the ESP8266 SoC is integrated with the TCP/IP protocol stack, enabling the microcontroller access to the Wi-Fi network and, hence, providing the possibility of transferring data toward cloud storage. The NodeMCU has programmability with LUA language or the Arduino Integrated Development Environment IDE [19]. The Arduino IDE software was selected as it provides a simplified integrated platform that allows the use of C or C++ language, while being able to run on any operating system. Sensors are small devices, having exceptional sensing capabilities for monitoring parameters such as temperature, humidity, light intensity, and soil moisture. Data obtained by the sensors are sent to the NodeMCU microcontroller board, where the data is processed, uploaded, and stored to a cloud database through the wireless network. The concept of IoT was born out of these remotely accessible sensor-based systems and storage [20].Raspberry Pi 4B is closed-source hardware that operates with open-source software, such as Linux or Pi OS. It has several connectivity options and is able to run multiple functionalities for a more complex IoT system. Raspberry Pi also provides wider possibilities when it comes to the field of video monitoring, and there is more room for future improvements, such as image acquisition or machine learning [21].

In the realm of environmental control for edible fungi, Bells were the first to adopt, in 1947, cultivation of mushrooms under environmentally controlled conditions, setting a precedent for the environmental control of edible fungi [22]. Kwon et al. designed an environmental control system using a heat pump in a variable opening chamber, which enabled an effective cultivation process, but the environmental parameters could not be monitored remotely in real-time [23]. Mahmud et al. built an automatic environmental monitoring system based on the ESP8266, along with a DHT11 temperature humidity sensor and an MQ135 carbon dioxide sensor. The system enabled users to remotely monitor the environmental condition through the ThingSpeak IoT platform [24]. Similarly, Subedi et al. developed a remote environmental monitoring system via NodeMCU, wherein measurements, such as those for temperature, humidity, soil moisture level and light intensity, could be accessed through the Blynk IoT platform [25]. However, the designs of Subedi et al. and Mahmud et al. lacked environmental control. Setiawati et al. used the Wemos D1 microcontroller, with DHT11, based on WSN and Blynk, to enable remote monitoring. Relays connected to the microcontroller regulated the temperature and humidity by turning the humidifier and exhaust fan on and off [26]. Suresh et al. demonstrated an automated climate control and monitoring system with an ATmega microcontroller, and effective temperature control was achieved by means of the thermoelectric cooler [26]. Nevertheless, the designs of Setiawati and Suresh et al. could not provide live stream image or video. Geng et al. combined Raspberry Pi and a microcontroller into the mobile greenhouse environment monitoring system so that parameters, such as temperature, humidity, and light intensity, and pictures, could be collected and uploaded to the webserver for remote access [27]. However, the system could not remotely control growth conditions since the control system depended on predetermined fixed threshold values.

As a result, the aim of this report was to design and develop an integrated IoT-based environmental control and monitoring system for home-based mushroom cultivation, where the growth conditions of the mushrooms could be remotely monitored and controlled through a mobile and web application. A camera module, as well as sensors that measure the temperature, humidity, light intensity, and soil moisture level are combined into the cultivation system’s growth requirements. Environmental control is enabled by microcontrollers and relays connected to the humidifier, thermoelectric coolers, and heater. The scope of the research included coding the NodeMCU boards and the Raspberry Pi 4B, in order for the sensors and camera unit to acquire specific data, images, or video throughout the cultivation process, while enabling remote environmental control by regulating relays connected to the microcontrollers and allowing remote monitoring and controlling of the system by connection with the Blynk IoT platform. The study also comprised investigation of the environmental behaviors within the cultivation chamber and validation of the effectiveness of the control system.

## 2. Methods and Materials

The development of the IoT based environmental control and monitoring system, as well as the cultivation of the mushrooms, were conducted in housing near the campus of the University of Nottingham Malaysia, Jalan Broga, 43500 Semenyih, Selangor, Malaysia, for a period of 4 months from January 2022 to April 2022.

### 2.1. Selection of Mushroom

The grey oyster mushroom was selected as the cultivation object among the edible mushrooms, due to the availability of prepared mushroom grow bags, the absolute ease of the cultivation process, the mushroom’s chemical tolerance and tolerance of a wide temperature range, as well as its ability to grow in a short period of time with high yield [28]. The mushroom growing bags used for the cultivation of the grey oyster mushroom were purchased from a supplier located at Pahang, Malaysia. The substrates of the growbags were formulated by a mixture of dried saw dust, rice bran and limestone in the mass ratio of 100:10:1, along with water added to the mixtures until a moisture content of 50% to 70% was reached. A quantity of 900 grams of the treated substrates, which was equivalent to 360 g of dried substrate, were packed into the plastic growing bag, sized at 90 mm in length and 80 mm in width. The substrates were compressed until a height of 20 cm was reached. The grow bag with mixed substrates was fitted with a plastic cover and neck. The hole in the plastic cover was filled with a little piece of sponge. After that, all of the growbags were pasteurised for 6 h at 121 °C and then cooled to room temperature. The spawns were then inoculated into the grow bags at a rate of 16 percent of the dry weight of the substrate, or 57.6 gram. Finally, all the bags were placed in an incubation chamber until the mycelia had completely colonized [29].

Oyster mushrooms can grow at moderate temperature and humidity, ranging from 18 °C to 30 °C [30], and 60% to 95%, respectively [10]. However, to obtain the optimum yield of the mushroom, the temperature and humidity must be controlled. The optimum temperature for the grey oyster mushroom during the mycelium stage is 28 °C [31], and the fruiting temperature should be kept around 20 °C to 25 °C. In addition, the development of the fruiting bodies is induced by changes in the temperature, especially a downshift of the temperature [6]. The ideal humidity level for the grey oyster mushrooms during the development of the mycelium and fruiting should encompass a range between 70%RH to 80%RH and 80%RH to 90%RH, respectively, while high humidity is favourable for pinning and fruiting [28]. However, as the development of the mycelium occurred within the mushroom grow bags, there was no monitoring and control of mycelial development in the cultivation chamber. As a result, the temperature and the relative humidity of the cultivation chamber were set to 25 °C and 90 %, respectively.

### 2.2. Design of the Mushroom Cultivation Chamber

**Figure 1:**
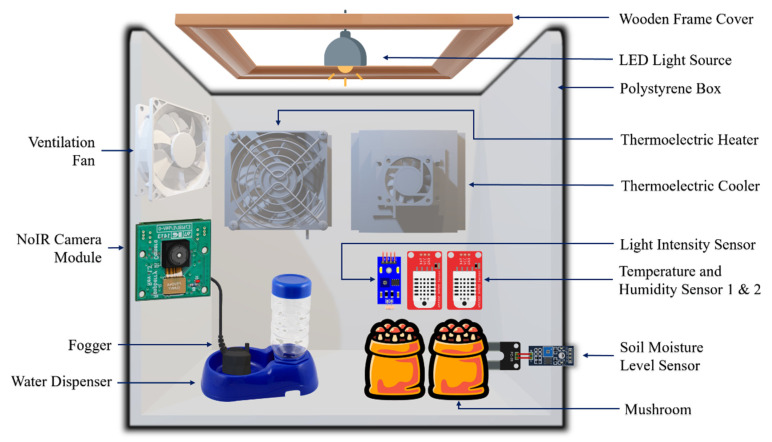
The design of the home-based mushroom cultivation chamber.

The design of the home-based mushroom cultivation chamber is presented in Figure 1. The mushroom cultivation chamber was based on a 42 Litre (0.042 m^3^) polystyrene box, with internal dimensions of 435 × 320 × 285 mm. A transparent plastic wrap, with internal dimensions of 500 × 400 × 10 mm, wrapped around the hollow wooden frame and served as a cover for the growth box, allowing the penetration of ab artificial light source, while protecting the mushrooms from contamination from physical, chemical, and biological sources. Despite light not being required during the mycelial development stage of the oyster mushroom, it is still a decisive factor in achieving a high yield of good quality mushrooms during the period of pinning and growth of fruiting bodies. Fruiting body development is affected by light intensity and the duration of light, based on the diurnal rhythm. Furthermore, it was found that oyster mushrooms grown under light of 200 lux has higher functional content compared with mushrooms exposed to a lower intensity of light. Furthermore, it was possible to decrease the duration of lightning while increasing the light intensity to ensure the amount of light needed to for fruiting. The morphological characteristics of the oyster mushroom, such as the cap size and stem length, have a positive correlation with light intensity, so an increase in the light intensity increases the cap size of the mushroom [32]. Therefore, an adjustable COB LED light was fitted at the top of the mushroom chamber to facilitate the proper light intensity, and the intensity of light was monitored by a light sensor.

The ventilation of the cultivation chamber was enabled by a 12 V fan located at the side of the polystyrene box, which constantly brought in the air from the chamber to the outside with a rate of 0.00463 m^3^/s. Underneath the ventilation fan is the 24 V fogger, which generates mist with a rate of 1.25 × 10^−7^ m^3^/s through the 20 mm ultrasonic mist ceramic disc. The fogger could increase the relative humidity in the chamber. The thermoelectric coolers and heater were fitted at the side of the box allowing precise control of temperature within the cultivation chamber. 

In addition, apart from the IoT sensors, a digital thermometer and a digital thermos-hygrometer were used to measure the temperature and the humidity of the cultivation chamber. The equipment needed per cultivation chamber included the following: 42 litre (0.042 m^3^) polystyrene box [490 × 365 × 330 mm]; wooden frame [560 × 460 × 10 mm]; transparent plastic wrap [30 m]; adjustable COB LED light [3.7 V]; 12 V DC brushless fan [40 × 10 × 40 mm]; automatic water dispenser [270 × 170 × 70 mm]; 24 V AC/DC adapter [AC 220 V/DC 24 V]; 24 V fogger [20 mm Disc]; DC power supply [12 V 20 A & 12 V 30 A]; digital LCD thermometer [−50~110 °C ± 0.1 °C]; digital LCD thermo-hygrometer [10~99%RH ± 3%RH) (−50~70 °C ± 1 °C]; TEC1-12706 Peltier thermoelectric cooler [12 V 6 A]; and TEC1-12706 Peltier thermoelectric heater [12 V 6 A].

### 2.3. IoT-Based Monitoring System

**Figure 2:**
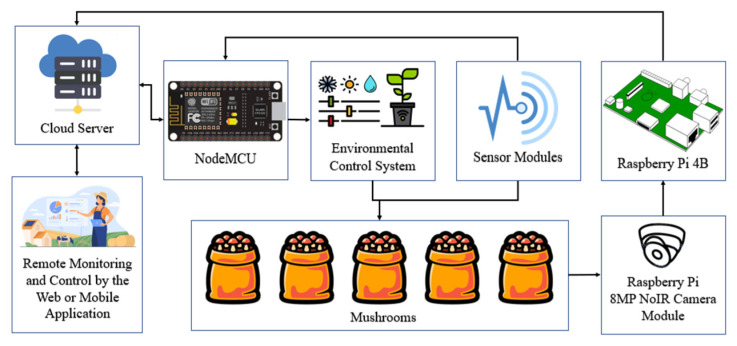
The overall block diagram for the IoT based environmental control and monitoring system.

Figure 2 represents the overall block diagram for the IoT-based environmental control and monitoring system. Several sensor modules, and a NoIR camera module, were connected to the NodeMCU and Raspberry Pi 4B, respectively, to ensure the proper monitoring of the farm while end data was sent the cloud server to provide remote monitoring.

#### 2.3.1. Live Video Monitoring and Image Capturing System

**Figure 3:**
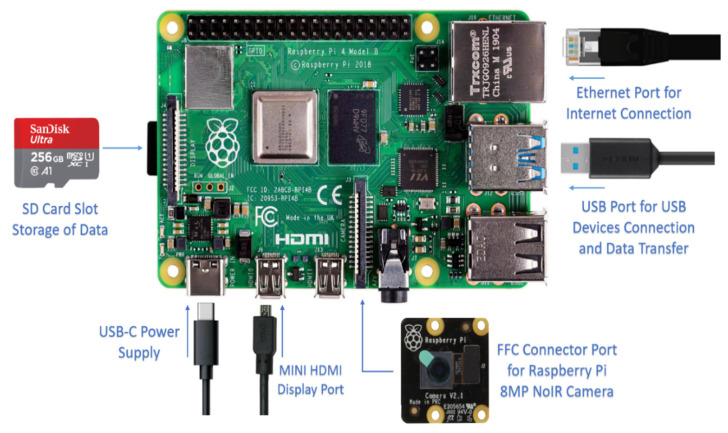
The setup of the Raspberry Pi 4B for live video monitoring and image capturing system.

To visualize the growth of the mushroom remotely, the NoIR camera module, connected to the Raspberry Pi 4B, provided live video of the mushroom through the WebSocket and Internet, while capturing images of the grey oyster mushrooms every 15 min and automatically uploading them toward the Dropbox, which enabled the user to remotely access and store the images of the mushrooms. Figure 3 shows the actual configuration for the Raspberry Pi 4B for the live video monitoring and image capturing system. The following equipment was needed: Raspberry Pi 4B, USB-C cable, USB power adapter, 128 G SD Card, HDMI cable, Mini HDMI to HDMI converter, flexible flat cable (2 m), NoIR camera module (8 MP), and Ethernet cable.

#### 2.3.2. Data Acquisition System

Parameters, such as temperature, humidity, light intensity, and soil moisture level, are crucial to the growth of mushrooms. Hence, enabling remote monitoring of those parameters could enhance the capability of the current equipment and enable recording of data for future analysis and reference. Three types of sensors were implemented in the environment monitoring system, namely, temperature and humidity sensor (DHT22) (https://www.cytron.io/p-dht22-sensor-module-breakout (accessed on 21 March 2022)), light intensity sensor (SN-LIGHT-MOD) (https://www.cytron.io/p-light-sensor-module (accessed on 21 March 2022)) and soil moisture sensor (SN MOISTURE-MOD) (https://www.cytron.io/p-moisture-sensor-module (accessed on 21 March 2022)). As the data was collected by the sensors, the NodeMCU was used to collect and process the data from the sensor for data plotting and the received data was then uploaded to the cloud server with the aid of the IoT platform Blynk. The users would then be able to remotely access real-time data through the web and mobile applications. Three NodeMCUs were used within the environment monitoring system. The first NodeMCU collected the light intensity, temperature, and the humidity within the cultivation chamber. Parameters such as soil moisture level, temperature, and humidity at different points of the cultivation chamber were gathered by the second NodeMCU. The third NodeMCU monitored the temperature and humidity outside the cultivation chamber. The equipment needed follows: NodeMCU Lua V3 ESP8266 Wi-Fi with CH340C, USB power adapter. Micro USB cable, 40 ways male to male jumper wire (30 cm), 40 ways male to female jumper wire (30 cm), 40 ways female to male jumper wire (30 cm), temperature and humidity sensor (DHT22) (0~100%RH ± 2%RH; -40~80 °C ± 0.5 °C), light intensity sensor (SN-LIGHT-MOD), Soil moisture sensor (SN MOISTURE-MOD), DHT22 sensor module breakout (SN-DHT22-MOD).

### 2.4. IoT-Based Environmental Control System

**Figure 4:**
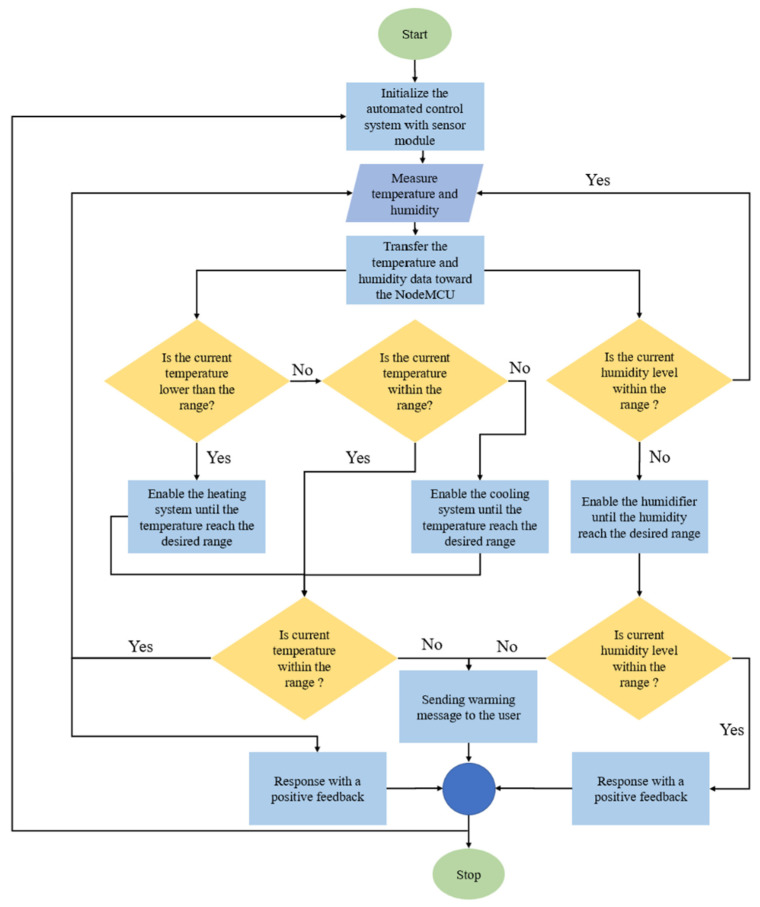
The flow chart of the automated temperature and humidity control system.

The environmental control system automated the thermoelectric cooler, thermoelectric heater and fogger within the cultivation chamber. The real-time data from sensor modules and the predetermined input data from the users were collected and processed within the NodeMCU, in order to initiate and govern the automated machine-to-machine interaction of all the installed electrical devices. For instance, to allow the automated control of temperature within the cultivation chamber, the desired temperature was provided into the system, the NodeMCU compared the input from the users with the real-time sensor temperature data and then manipulated the thermoelectric cooler and heater by means of the relays. Similarly, the relative humidity level within the cultivation chamber was automated by comparing the threshold given with the real-time sensor data to govern the operation of the fogger. Figure 4 illustrates the working principle of the automated temperature and humidity control system.

**Figure 5:**
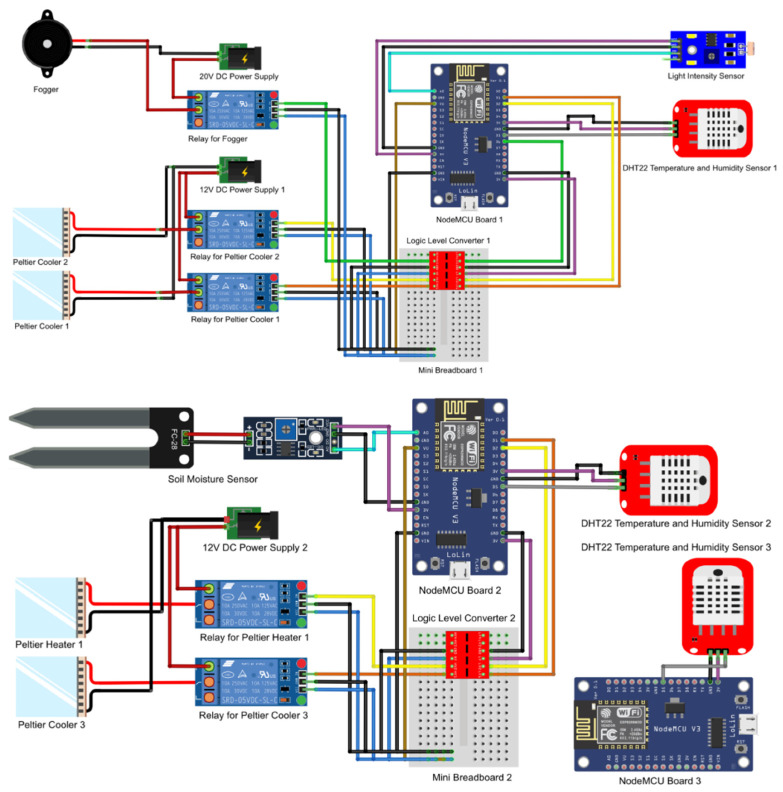
The actual schematic diagram for the NodeMCU board 1, 2 and 3.

As mentioned, the temperature threshold and the relative humidity threshold for cultivation of grey oyster mushrooms was set at 25 °C with a range of 0.5 °C, and 90%RH with a range of 2%RH, respectively. Hence, the condition of the cultivation chamber was maintained within a range of 24.5 °C~25.5 °C and 88%RH~92%RH. If the DHT 22 sensor measured a temperature value which was higher than 25.5 °C, the data would be processed by the NodeMCU microcontroller to activate the cooling system by sending the electrical signal to the relays. Conversely, if the sensor picked up a temperature lower than 25.5 degrees Celsius, the microcontroller would instruct the relay to switch on the heater. Furthermore, when the sensor detected a reduction in RH below 88%, data was transferred to the microcontroller, which ordered the relay to switch on the humidifier. If relative humidity exceeded 92 percent, the microcontroller would command the relay to switch off the humidifier. There were 3 NodeMCUs used within the environmental monitoring system; however, only the first two boards were used in the automated temperature and humidity control system. The first NodeMCU controlled the relays for the 2 Peltier coolers and the fogger and the second NodeMCU controlled a Peltier cooler and a thermoelectric heater. Both the NodeMCUs required a logic shifter in order to amplify the 3.3 V signal to 5 V to the relays. The equipment needed were the following: 4-Channel logic level Shifter (BB-4C-LSHIFT), Single channel 5 V relay breakout board (BB-RELAY-5V-01), 40 ways male to male jumper wire (30 cm), 40 ways male to female jumper wire (30 cm), 40 ways female to male jumper wire (30 cm), and Bread board Mini (35 mm × 47 mm) (BC-BB-MW). Figure 5 illustrates the actual schematic diagram for the NodeMCU boards 1, 2 and 3.

### 2.5. Web and Mobile Application

As the entire IoT based environmental control and system started with the acquisition of specific data from different sensors and the camera unit, the control algorithm enabled the automated control system to take appropriate actions, based on the data obtained from the sensors and the user input, to monitor and control the environmental conditions within the cultivation chamber. The data collected from the sensors and camera module were then sent to the cloud to enable remote monitoring. The system was developed with the aid of Arduino IDE, Geany and Thonny.

Monitoring the data acquired from the sensors and controlling the automated environment were enabled through the Blynk IoT platform. There was a total of 10 data streams were used, covering temperature, humidity, light intensity, soil moisture level, threshold temperature and threshold humidity. Each data stream was assigned a unique UID, and the API keys were used to write and read data from the server. The data from the sensors was written to the Blynk IoT platform through the ESP8266 Wi-Fi module and the thresholds given by the user sent to the NodeMCU through the Blynk IoT platform. The data stored in the Blynk IoT platform could be remotely accessed at any time via Wi-Fi connection. The data could also be exported in the format of Comma-Separated Values (CSV) format for storage or further analysis. The web dashboard and mobile dashboard were created to ease the monitoring and controls for the cultivation chamber. The coding for the three NodeMCU boards are shown in Appendix A.

In terms of visualizing the growth of the mushrooms remotely, the JSMpeg, a JavaScript-based video player, loaded the static videos via Ajax and allowed low latency video streaming (~50ms) through the Web Sockets. The pictures of the mushrooms were obtained by making screenshots of the live broadcast every 15 min, and automatically uploading to the Dropbox through the aid of Dropbox API, which enabled the user to access and store the images of the mushrooms remotely. The detail of coding is shown in Appendix A.

**Figure 6:**
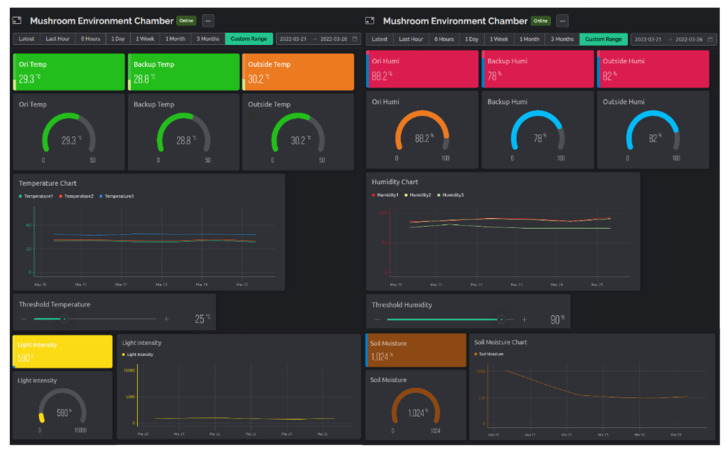
The user interface of the web dashboard presenting the real time status of different conditions in the mushroom cultivation chamber.

**Figure 7:**
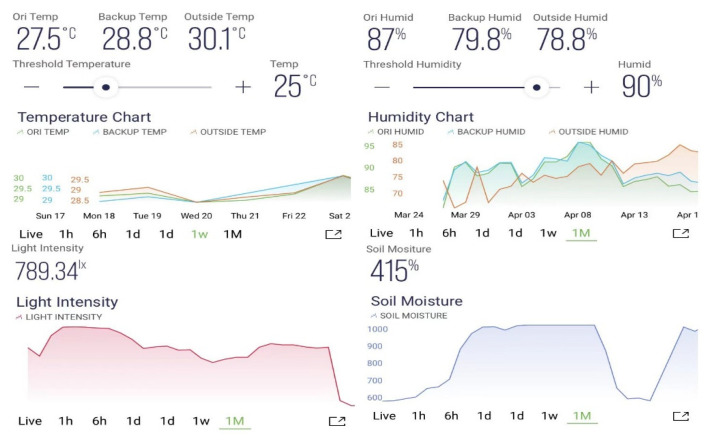
The user interface of the application dashboard presenting the real time status of different conditions in the mushroom cultivation chamber.

Figure 6 and Figure 7 shows the user interface (UI) of the web dashboard and application dashboard, presenting the real time status of the mushroom cultivation chamber, respectively. The growth conditions, such as temperature, humidity, light intensity and soil moisture, were monitored using this interface.

### 2.6. Analysis

After the configuration of the hardware and software to the IoT-based environmental control and monitoring system, several experiments were carried out to determine the performance of each sensor. The data were recorded and compared to laboratory instruments. The R^2^ value and the mean absolute percentage error (MAPE) of each sensor was calculated. The correlation between the actual data and the sensor-measured data was determined by the R square value and the equation used for calculation of R^2^ is shown in Equation (1) [33]:

(1) $$R^{2}=1-\frac{\sum_{1}^{n}{\left(D_{act}-D_{pre}\right)}^{2}}{\sum_{1}^{n}{\left(D_{act}-{\bar{D}}_{act}\right)}^{2}}$$

where $D_{act}$ is actual variable, $D_{pre}$ is predicted variable, ${\bar{D}}_{act}$ is the mean value of the actual variable

The error of the actual and measured data from the control and monitoring system sensor was investigated. The equation used for the mean absolute percentage error (MAPE) calculation of the sensor during the calibration process is shown in Equation (2) [34]:

(2) $$MAPE=\frac{100}{n}-\overset{n}{\underset{i=1}{\sum }}\left|\frac{D_{pre}-D_{act}}{D_{act}}\right|$$

where $D_{act}$ is Actual variable, $D_{pre}$ is Predicted variable.

#### 2.6.1. Light Intensity Sensor (SN-LIGHT-MOD)

With 0.5 m between the light sources and the light intensity measuring instrument (BF05), the light intensity of the adjustable COB LED light was tuned to 0 lux, 100 lux, 200 lux, 300 lux, 400 lux, 500 lux and 1000 lux, and the response of the light intensity sensor examined.

#### 2.6.2. Soil Moisture Sensor (SN MOISTURE-MOD)

The soil moisture sensor (Cytron Technologies, Malaysia) was calibrated with the Soil Moisture Meter (Gouevn, China), where several samples of soil were measured to check the accuracy of the sensor. The values obtained from the soil moisture sensor was compared to the Soil Moisture Meter MLX2.

#### 2.6.3. Temperature and Humidity Sensor (DHT22)

Sensor calibration was conducted to determine the accuracy of the sensor in reading the fluctuation of temperature, determined by senor calibration. The temperature and humidity sensors and the LCD digital thermohydrometer were placed in the cultivation chamber. The values obtained from the DHT22 sensor were compared to those of the digital thermo hygrometer.

## 3. Results and Discussion

### 3.1. Light Intensity Sensor

Table 1 shows a comparison of light intensity measured by the light intensity measuring instrument (BF05) and the light intensity sensor (SN-LIGHT-MOD). The average absolute error of the light intensity measured by the light sensor SN-LIGHT-MOD was 29.83 lux, the minimum absolute error was 13 lux, and the minimum absolute percentage error was 4%. The maximum absolute error was 65 lux and the maximum absolute percentage error was 13%. The mean absolute percentage error was calculated as 7.82%, which was less than 10%. Generally, a highly accurate forecasting was enabled if the MAPE was less than 10%, and if the MAPE value lay between 10~20% the system had good forecasting ability.

### 3.2. Soil Moisture Sensor

The soil moisture sensor (SN MOISTURE-MOD) consisted of two conducting probes that measured the soil moisture content based on the change in resistance between the two conducting probes. The amount of moisture within the soil was inversely proportional to the resistance between the two conducting probes, and, hence, if the moisture content within the soil increased, the resistance between the two probes decreased. An analog to digital converter (ADC) was used to process the analog output from the soil moisture sensor, where the output of the sensor varied over an ADC value range of 0 to 1024. As a result, to obtain the soil moisture level in percentage a conversion formula was needed:

(3) $$Moisture\;in\;percentage=100\%\;-\left(\frac{ADC\;Value}{1024}\times 100\%\right)$$

Table 2 shows a comparison of soil moisture level measured by the soil moisture sensor (SN MOISTURE-MOD) and the soil moisture meter MLX2. The average absolute error of the soil moisture level measured by the soil moisture level (lux) was 0.7%, the minimum absolute error was 0.5% and the minimum absolute percentage error was 1.67%. The maximum absolute error was 1.2% and the maximum absolute percentage error was 11.01%. The mean absolute percentage error was calculated as 4.21%, which was less than 10%. Hence, highly accurate forecasting was enabled.

### 3.3. Temperature and Humidity Sensor

Table 3 shows a comparison of the temperature and humidity measured by the thermo hygrometer and the temperature and humidity sensor (DHT22). Treating the thermohydrometer as a reference point. the average absolute error of temperature measure by the DHT22 was 0.33 °C. The maximum absolute error was 0.6 °C, while the minimum absolute error of temperature was 0.2 °C. The maximum absolute percentage error was 2.28%, while the minimum percentage error of temperature was 0.73%. where the mean absolute percentage error (MAPE) was calculated as 1.22%. On the other hand, the average absolute error of humidity measure by the DHT22 was 1.73%RH. The maximum absolute error was 3.0 %RH, while the minimum absolute error of temperature was 0.47%RH. The maximum absolute percentage error was 3.39%, while the minimum percentage error of temperature was 0.55%. where the mean absolute percentage error (MAPE) was calculated as 1.73%, which was less than 10%. Thus, highly accurate forecasting was enabled.

### 3.4. Performance of the Environmental Monitoring and Control System

**Figure 8:**
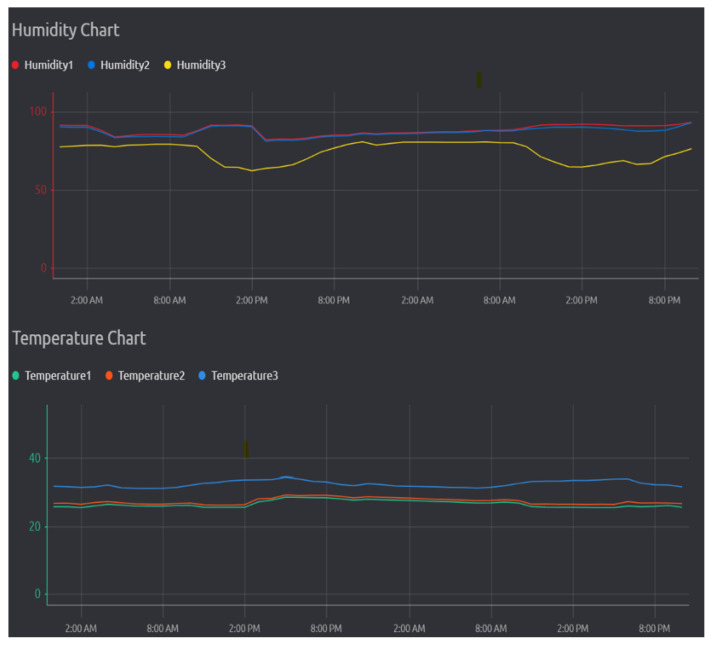
System generated humidity chart and temperature chart for the mushroom cultivation chamber.

The comparison results of temperature and relative humidity for 2 days between control and non-controlled environments are shown in Figure 8. Humidity 1 and 2 were the sensors located within the cultivation chamber and temperature 3 was the sensor located in the uncontrolled environment. The maximum humidity outside the non-controlled chamber environment ranged from 88.5%RH °C to 59.07%RH and varied due to weather precipitation. This work also illustrates successful control of the humidity as the humidity within the controlled environment ranged between 91.01%RH to 88.59%RH, whereas there was a drop of the humidity level at the first 3 am and the first 2 pm, due to water depletion within the automatic water dispenser system. Temperature 1 and 2 and the sensor remained within the cultivation chamber and temperature 3 was the sensor that was located in the uncontrolled environment. The maximum temperature outside the non-controlled chamber environment ranged from 32.59 °C to 29.88 °C, and varied between day and night. Figure 8 illustrates successful control as the temperatures within the controlled environment ranged between 24.4 °C and 26.3 °C. The increase of the temperature at the second 2 pm was a consequence of water level depletion, requiring opening the lid for water refill.

### 3.5. Performance of the Video and Image Monitoring System

**Figure 9:**
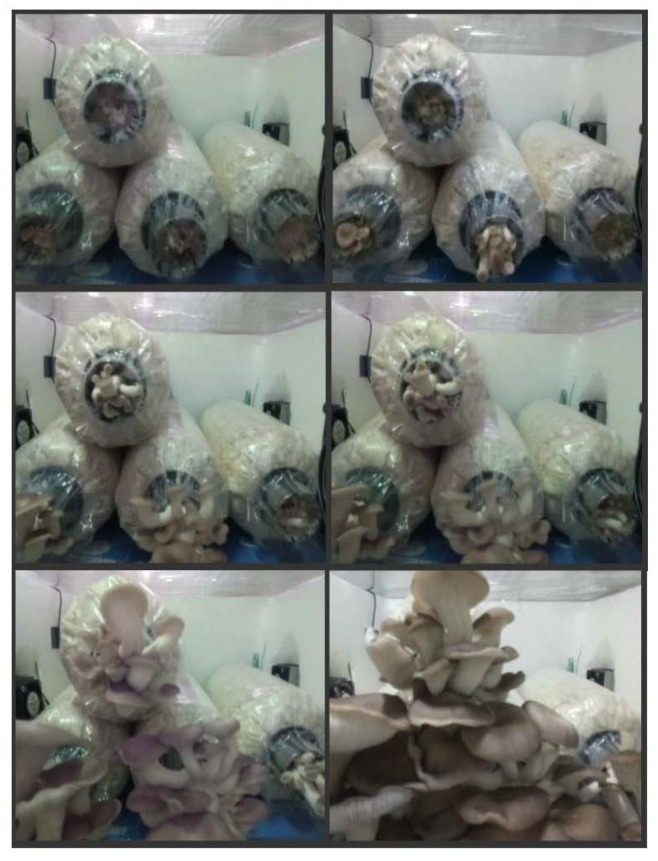
The growth of the grey oyster mushrooms monitored by the camera. From left to right and from top to bottom for the stage of increasing growth stage.

The images of the mushrooms were successfully uploaded through Dropbox, and the growth of the mushroom could be monitored, Figure 9 shows the transition of the growth of the grey oyster mushroom, when the photo was captured at 8am every day and uploaded to the Dropbox automatically. As a result, the system enabled the user to quantify the growth of the mushrooms remotely and machine learning could be applied in the future to determine the time for harvesting.

## 4. Conclusions

The development of the IoT-based environmental control and monitoring system, as well as the cultivation of mushrooms were successfully implemented in this work. The system enables the user to monitor and control temperature, humidity, light intensity, and soil moisture level within the cultivation chamber through a mobile and web application, Blynk, while the user is able to visualize the growth of the mushroom remotely by video and images online. The accuracies of the four different sensors were compared with their respective laboratory instruments, where the data was processed through calculation, such as mean absolute percentage error (MAPE) and R square value. The results showed that all the sensors were accurate, and the readings were close to the actual conditions. In addition, the IoT-based environmental control and monitoring system developed was able to keep parameters such as humidity and temperature under control throughout the cultivation process. Image processing schemes were also applied to quantify the growth of the mushrooms during the fruiting phase. Four types of parameters were implemented throughout the cultivation process. Sensors, such as a carbon dioxide sensor or oxygen level sensor are recommended to be implemented into the system, and further research can focus on the performance of these sensors. The implementation of IoT in the cultivation of mushrooms would help the mushroom industry in receiving real-time data, as well as facilitating responses to any severe changes throughout the cultivation process, and hence, resulting in higher yields.

## Figures and Tables

**Table 1:** Comparison of light intensity between BF05 lux meter and (SN-LIGHT-MOD) light sensor.

Light Intensity from Light Intensity Measuring Instrument BF05 (lux)	Light Intensity from the Light Sensor SN-LIGHT-MOD (lux)	Absolute Error (lux)	Absolute Percentage Error (%)
100	87	13	13.00
200	213	13	6.50
300	281	19	6.33
400	416	16	4.00
500	447	53	10.60
1000	935	65	6.50

**Table 2:** Comparison of soil moisture level between MLX2 soil meter and the soil moisture level sensor.

Soil Moisture Level from MLX2 Soil Meter (%)	Soil Moisture Level from Soil Moisture Level Sensor (%)	Absolute Error (%)	Absolute Percentage Error (%)
22.3	21.8	0.5	2.24
23.9	23.5	0.4	1.67
24.6	23.8	0.8	3.25
10.9	9.7	1.2	11.01
27.4	28.1	0.7	2.55
19.2	19.6	0.4	2.08
13.5	14.4	0.9	6.67

**Table 3:** Comparison of the temperature and humidity between thermo hygrometer and the DHT22 sensor.

**Temperature from the Thermo** **Hygrometer (°C)**	**Temperature from the DHT22 Sensor (°C)**	**Absolute Error (°C)**	**Absolute Percentage** **Error (%)**
26.6	26.4	0.2	0.75
27.1	26.6	0.5	1.85
26.1	25.9	0.2	0.77
26.3	25.7	0.6	2.28
27.5	27.7	0.2	0.73
30.2	29.9	0.3	0.99
25.8	26.1	0.3	1.16
**Humidity from the Thermo** **Hygrometer (%RH)**	**Humidity from the DHT22** **Sensor (%RH)**	**Absolute Error (%RH)**	**Absolute Percentage** **Error (%)**
86.1	85.63	0.47	0.55
87.1	88.4	1.3	1.49
78.1	80.1	2	2.56
67.2	65.6	1.6	2.38
70.9	73.1	2.2	3.10
88.6	91.6	3	3.39
90.1	91.65	1.55	1.72

## Data Availability

Not applicable.
